# Scalable Fabrication of Quasi-Three-Dimensional Chiral Plasmonic Oligomers Based on Stepwise Colloid Sphere Lithography Technology

**DOI:** 10.1186/s11671-015-1102-1

**Published:** 2015-10-18

**Authors:** Shiwei Xie, Jinzhe Yang, Xiao Xiao, Yidong Hou, Jinglei Du, Lin Pang, Xie Li, Fuhua Gao

**Affiliations:** Department of Physics, Sichuan University, Chengdu, 610064 China; College of Science, China Three Gorges University, Yichang, 443002 China; Key Laboratory for High Energy Density Physics and Technology, Ministry of Education, Sichuan University, Chengdu, 610064 China; Department of Physics, Key laboratory of Micro- and Nano-Photonic Structures (Ministry of Education) and State Key Laboratory of Surface Physics, Fudan University, Shanghai, 200433 China

## Abstract

We report a simple and scalable method for the fabrication of spiral-type chiral plasmonic oligomers based on the stepwise colloid sphere lithography technology. Through carefully adjusting the azimuthal angle *Φ* of polystyrene (PS) sphere array monolayer and the deposition thickness *k*_*n*_, the chiral plasmonic oligomers composed of four achiral particles can be successfully fabricated on a desired substrate. And their chiral sign, i.e., left-hand or right-hand, is dependent on the anticlockwise or clockwise deposition sequence of the achiral particles. The measured results show a large chiroptical resonance in the visible region, and this resonance can be easily adjusted by using different sizes of PS spheres. Our in-depth theoretical and experimental researches further reveal that the obtained chiral plasmonic oligomers are indeed a kind of quasi-three-dimensional chiral nanostructures, which own a three-dimensional geometrical morphology, but with nonreciprocity chiroptical effect. The ease and scalability (>1 cm^2^) of the fabrication method make chiral plasmonic oligomers promising candidates for many applications, such as chiral biosensor and catalysis.

## Background

According to Lord Kelvin, any geometrical figure or group of points is defined as chiral if its mirror image cannot be made to coincide with itself [1]. Chirality is ubiquitous in nature and plays a crucial role in the living body. Almost all of the important biomolecules, such as proteins, amino acids, and DNA, own the same chirality, i.e., left-hand (LH) and right-hand (RH). And distinguishing the chirality of molecules becomes very significative for life. Although enantiomers (i.e., left-handed (LH) and right-handed (RH) substances) possess the same physical and chemical properties, they usually interact differently with left- and right-circularly polarized light (LCP and RCP). In fact, the cross coupling between the electric and magnetic dipoles in the chiral medium can break the degeneracy between the helicity eigenmodes of light (i.e., LCP and RCP), leading to an absorption difference (i.e., circular dichroism, CD) or a phase retardation difference (i.e., circular birefringence, CB) for the LCP and RCP. This measurable chiroptical effect paves a way to achieve the structural characterization and spectroscopy of chiral molecules and manipulate the polarization state of light. However, the magnetoelectric coupling in the natural chiral molecules, such as DNA, proteins, amino acids, and viruses, is very weak [2]. This limits the ability of practical applications for the polarization manipulation and high-sensitivity sensing.

Recently, chiral plasmonic structures (CPS) have been demonstrated to own giant chiroptical effect [3] and attracted many attentions. These CPS cannot only be used to enhance the chiroptical effect of chiral molecules and improve the detection sensitivity [4, 5] but also be used to achieve some amazing physical phenomena, such as negative refraction [6, 7], repulsive Casimir force [8], and spin Hall effect [9]. These remarkable properties make CPS a very popular metamaterial in many fields, such as biology, pharmacology, and nanophotonics. Typically, the currently developed CPS can be divided into two categories: one is the two-dimensional (2D) CPS. This type of CPS owns a plane-like geometrical morphology, and the twist sense of their structures is reverse when looked from the opposite side, such as a gammadion structure [10], L-shaped structure [11], and rose-shaped structure [12]. The chiroptical effect from these CPS is nonreciprocal, which is similar to the Faraday effect [13]. The other is the three-dimensional (3D) CPS. The twist sense of this type of CPS is the same when looked from the opposite side, such as Helix [14], twisted arcs [3, 15], Y-shaped [16], U-shaped [17], cross-shaped [18], and three-dimensional triple-helical nanowires [19, 20]. The chiroptical effect from these CPS is reciprocal, just like that from natural light active medium [21]. However, the fabrication of these 2D or 3D CPS usually relied on the electron beam lithography technique [22], direct laser writing technique [23], and so on. The process technology usually is time-consuming, expensive, and small area limited, which limited the related research and applications.

Molecule assembly techniques, such as DNA origami technique [24, 25], have been demonstrated to a kind of efficient and low-cost method for the fabrication of CPS. The chiroptical resonance from the CPS is randomly dispersed in solvent usually located in the visible region. Glancing angle deposition technique is another low-cost, material-independent, and parallel fabrication technique for the fabrication of CPS. With the help of various masks, such random nanohole array [26], square nanoparticle array [27], and aligned ZnO nanorods [28], various CPS with chiroptical resonance located in the near-infrared and visible region have been achieved. Particularly, our previous work also demonstrates that with the help of assembled polystyrene (PS) sphere array, a kind of shell-like chiral nanostructures can be achieved [29]. This kind of CPS can combine the magnetic or dielectric spheres to form chiral “Janus particles” [30] or “patchy particles” [31, 32] and will find potential applications in the colloid field. However, the cancellation effect originated from the PS array limited the related applications. In this paper, we propose a new method for the fabrication of CPS based on stepwise colloid sphere lithography technology, which is quasi-three-dimensional chiral plasmonic oligomers (CPO) composed of four achiral particles. Furthermore, we systematically analyze the optical properties of our fabricated CPO with the help of full-wave simulation and the measurement of CD spectra and optical activity. And the results reveal the reciprocal chiroptical effect of our fabricated CPO.

## Methods

**Fig. 1:**
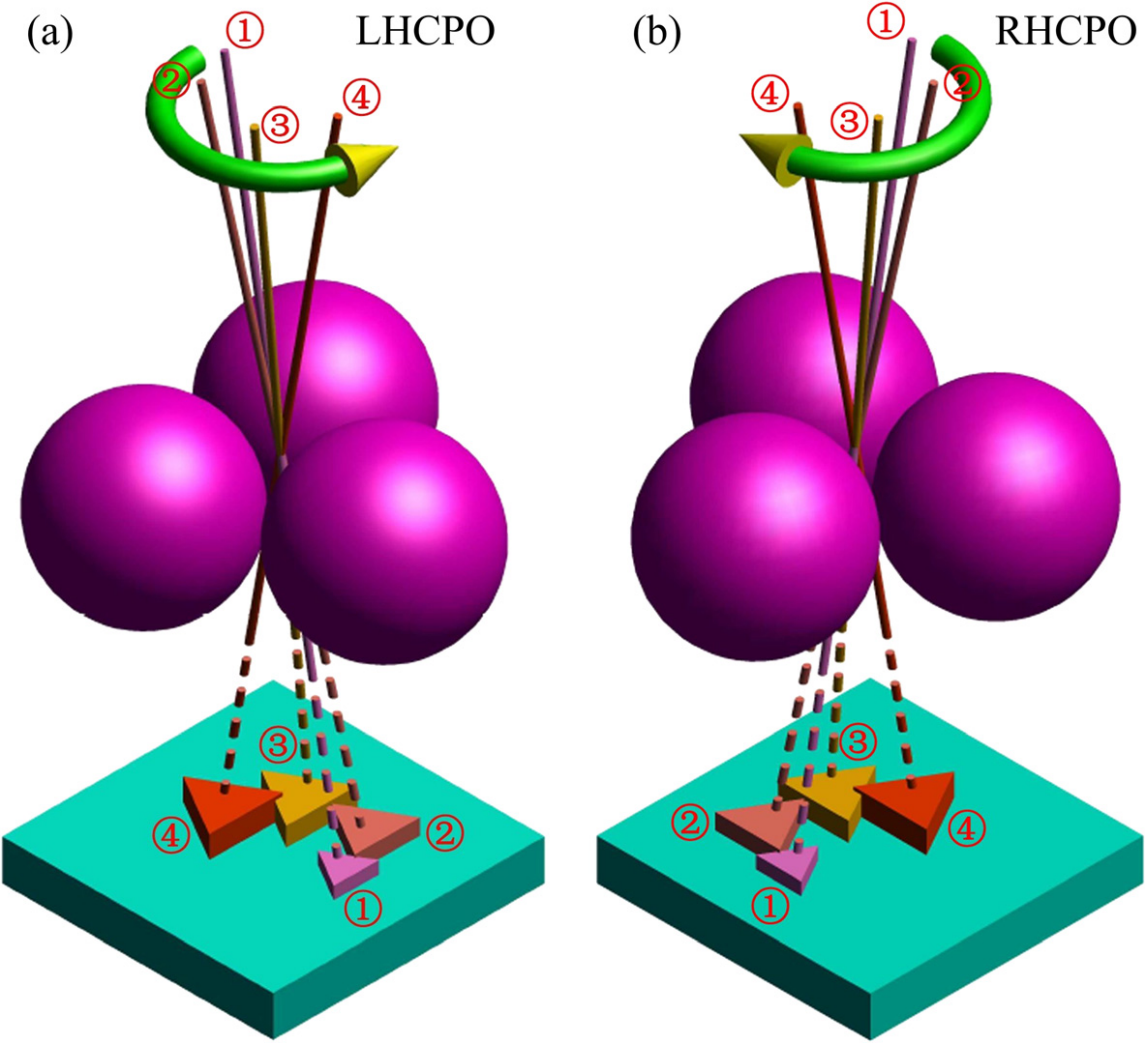
Design principle schematic of LHCPO (**a**) and RHCPO (**b**). The numbers ①, ②, ③, and ④ denote the *n*-th material deposition step and the formed achiral particles, and the *green curves with yellow arrow* denote the deposition sequence, where the anticlockwise deposition sequence refers to LHCPO and the clockwise deposition sequence refers to RHCPO

This section describes the design principle and methods of the CPO fabricated. Colloid sphere lithography technology is a powerful method for the fabrication of nanostructures. And a great number of nanostructures have been obtained by this method, such as triangle particle array [33], binary array [34], and multiplex quasi-3D grids [35]. The basic principle of this technology is the use of a PS array as mask, and the morphology and size of the obtained nanostructures are dependent on the projection of gap between PS spheres with respect to material vapor beam [36]. In this work, we employ this technology to arrange four achiral nanoparticles with different sizes in a concentric circle, and in this case a kind of new CPS, i.e., CPO, can be formed on substrate, as shown in Fig. 1a, b. The left-handed chiral plasmonic oligomers (LHCPO) are related to the anticlockwise deposition sequence (Fig. 1a), while the right-handed chiral plasmonic oligomers (RHCPO) are related to the clockwise deposition sequence (Fig. 1b). In order to obtain CPO with high optical chirality and geometrical chirality, the size and position of the four achiral particles should be properly adjusted in the fabrication process.

**Fig. 2:**
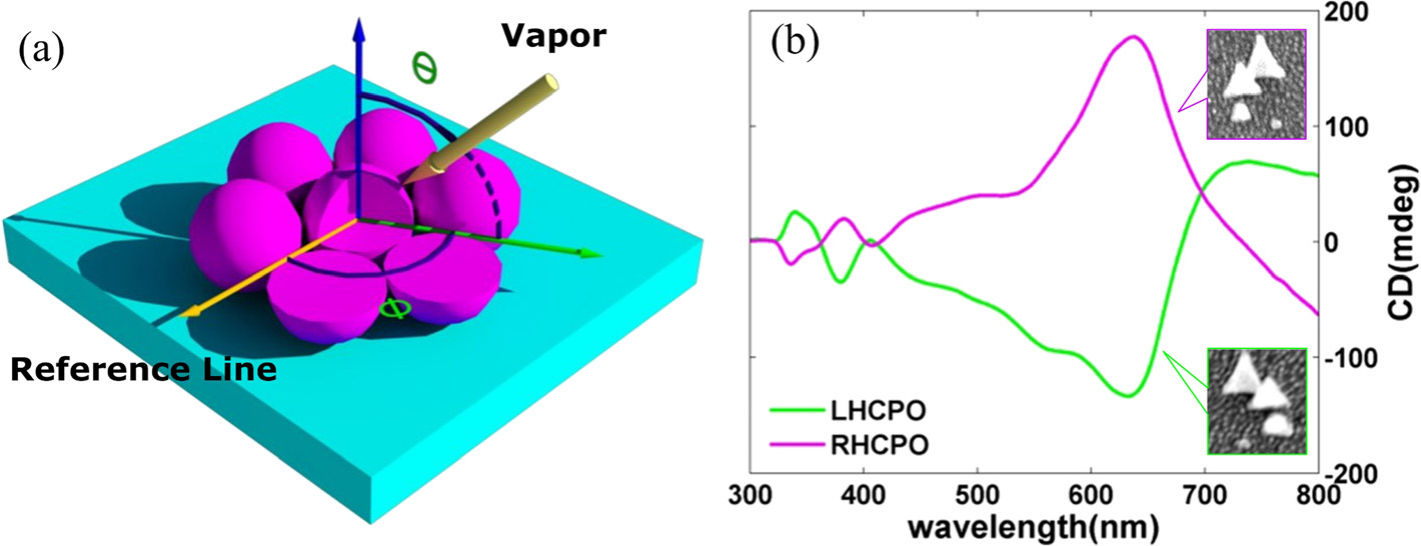
**a** Schematic diagram of material deposition on assembled PS array monolayer. Where *θ* is the deposition angle, which is defined as the material vapor beam with respect to the substrate normal, and *Φ* is the azimuth angle with respect to the PS array monolayer. **b** The measured CD spectra of LHCPO (*green*) and RHCPO (*aubergine*) with *θ* = 20°, *k*_1, 2, 3, 4_ = 8, 25, 40, and 55 nm, respectively, and *ΔΦ* = ±60°. The *insets* are the related CPO SEM images

Figure 2a is the material deposition schematic diagram, where *θ* is the deposition angle, which is defined as the material vapor beam with respect to the substrate normal, and *Φ* is the azimuth angle with respect to the PS array monolayer. In the formation of CPO, the size of the achiral particles and the diameter of the circle where the achiral particles are located are dependent on the deposition angle *θ*, and the position is dependent on the azimuth angle *Φ*. The deposition thickness *k* is another important parameter and defined as the deposition thickness on the plane perpendicular to the vapor beam. In general, the height of the achiral particles is dependent on the deposition thickness *k.* However, due to surface energy minimization [27], the deposition thickness *k* will indirectly affect the size of achiral particles and result in great contraction of the achiral particles with small deposition thickness. In the fabrication of CPO, these influence factors should be considered. The inset scanning electron microscope (SEM) images shown in Fig. 2b are the obtained CPO with *θ* = 20°, *k*_1, 2, 3, 4_ = 8, 25, 40, and 55 nm, and *ΔΦ* = ±60°, where *k*_*n*_ is the deposition thickness in the *n*-th step material deposition process, *ΔΦ* is the relative azimuth angle between the approaching two-step material deposition processes, and the signs “+” and “−” refer to the anticlockwise deposition sequence (i.e., LHCPO) and the clockwise deposition sequence (i.e., RHCPO). The measured CD spectra show that the LHCPO and RHCPO nearly own opposite CD spectra and a maximum CD signal of ~180 mdeg is obtained at 637 nm. These experiment results indicate the effectiveness of the method proposed in this paper. Due to the predominant fabrication method, the CPO with area of >1 cm^2^ can be easily obtained. These features will make CPO a promising candidate for many applications, such as chiral biosensor and catalysis.

**Fig. 3:**
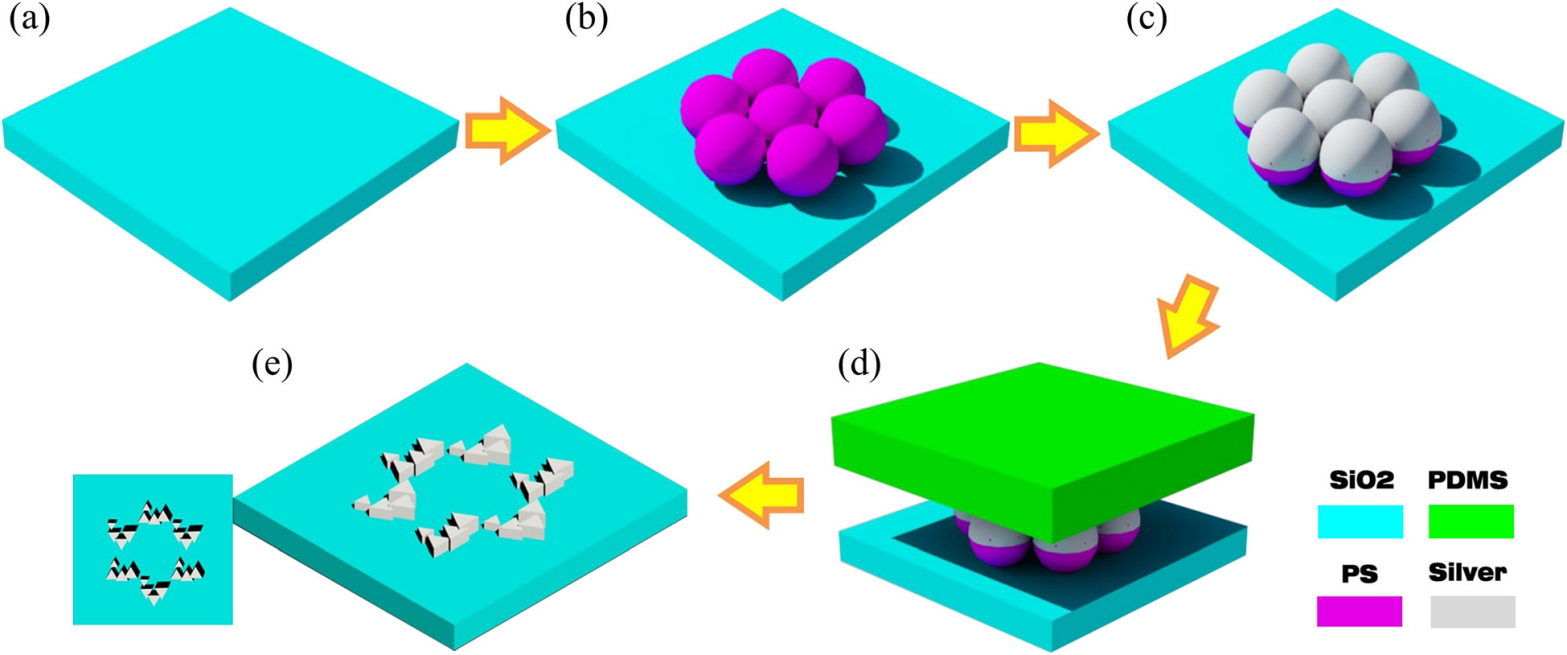
Schematic diagram of the preparation process. **a** BK7 glass substrate. **b** Self-assembly of polystyrene microspheres. **c** Glancing angle deposition of metal vapor. **d** Removal of unwanted portions with PDMS. **e** Resulting structure and its top view

The quasi-3D CPO are fabricated on a BK7 glass substrate through four-step Ag deposition on a monolayer of PS array. The detailed fabrication schematic diagram is shown in Fig. 3.

Firstly, a BK7 glass is used as substrate. Before being used in the micro-sphere-assembled process, the BK7 glass substrate is immerged in a mixed solution of sulfuric acid/hydrogen peroxide (3:1) for more than 1 h at 80 °C. Then, the substrate is rinsed in deionized water for several times and immerged in another mixed solution of ultrapure water/ammonium hydroxide/30 % hydrogen peroxide (5:1:1) for 1 hour under ultrasonication, sequentially. Finally, the substrates were thoroughly rinsed with deionized water and stored under deionized water until required. Secondly, a monolayer of PS array with hexagonal symmetry is obtained on the BK7 glass substrate through the method reported in the literature, as shown in Fig. 3b. The PS spheres with a diameter of 750 nm and weight concentration of 10 % are purchased from Thermo Scientific Company and used without further treatment. Thirdly, the Ag deposition on the closely packed PS array monolayer is performed inside a vacuum thermal evaporation system, as shown in Fig. 3c. The base pressure, the deposition rate, and the temperature are 4 × 10^−4^ Pa, 1 nm/s, and 30 °C, respectively. For a single-step deposition, the deposition time needs to be precisely controlled (i.e., deposition thickness *k*), together with the angles *θ* and *Φ*, which can be easily adjusted by tilting and rotating the samples with the improved slide holder in the vacuum chamber, respectively. For stepwise depositions, the operational process is the repeat of the single deposition. All the deposition angle *θ* is set to 20°, *ΔΦ* is set to ±60^o^ for LHCPO (i.e., +60°) and RHCPO (i.e., −60°), and the deposition thickness *k*_1, 2, 3, 4_ is set to 8, 25, 40 and 55 nm, respectively. Finally, a polydimethylsiloxane (PDMS) stamp is used to remove the silver-coated PS array monolayer. As shown in Fig. 3d, e, the PDMS stamp is placed on the surface of the silver-coated PS array monolayer with a uniform pressure of about 500 g/cm^2^. After being lifted up, the PS array monolayer is transferred to the surface of the PDMS stamp, due to the larger Van der Waals force from the PDMS stamp when compared with the silica substrate. When all of the process is finished, a kind of quasi-3D CPO is achieved on the BK7 glass substrate.

## Results and Discussion

**Fig. 4:**
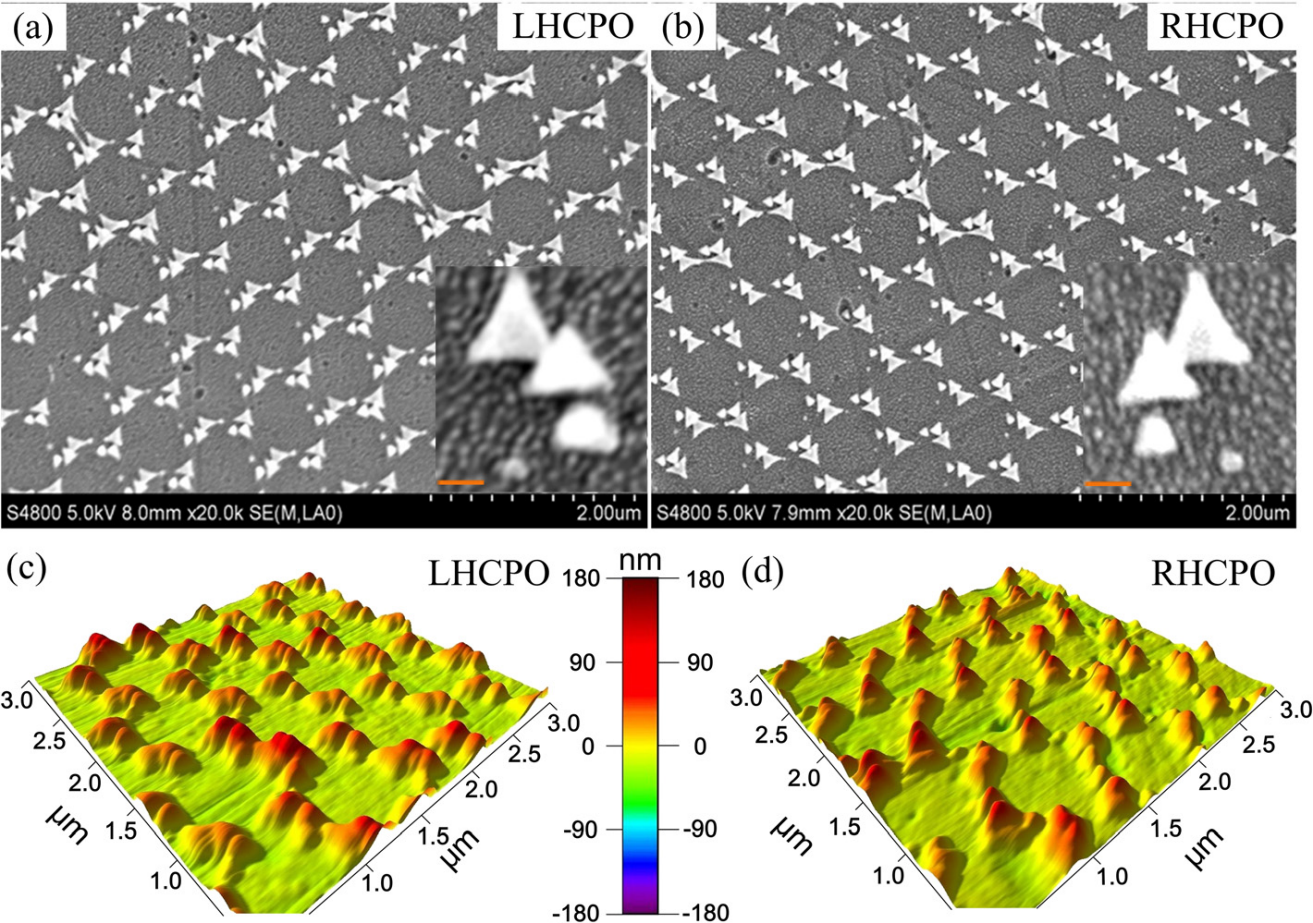
The measured SEM and AFM images of LHCPO and RHCPO with *θ* = 20°, *k*_1, 2, 3, 4_ = 8, 25, 40, and 55 nm, respectively, and Δ*Φ* = ±60°. **a**, **c** From LHCPO. **b**, **d** From RHCPO. The *insets* in **a** and **b** are the enlarged images of one CPO

The SEM images are collected by the Hitachi S-4800 SEM. Figure 4a, b shows the SEM images of the obtained LHCPO and RHCPO, respectively. And the insets are the enlarged SEM images of single CPO. From these images, we can get that the CPO is well formed on the BK7 glass substrate. Obviously, the chiral sign (i.e., LH or RH) is dependent on the anticlockwise or clockwise deposition sequence. The Nikon Eclipse Ti-U atomic force microscopy (AFM) also has been used to verify the 3D geometric morphology of the quasi-3D CPO, and the obtained AFM images are shown in Fig. 4c, d, where the 3D geometric morphology is well presented. The deposition thickness in every material deposition step is also detected by AFM, and the results meet well with that designed thickness.

**Fig. 5:**
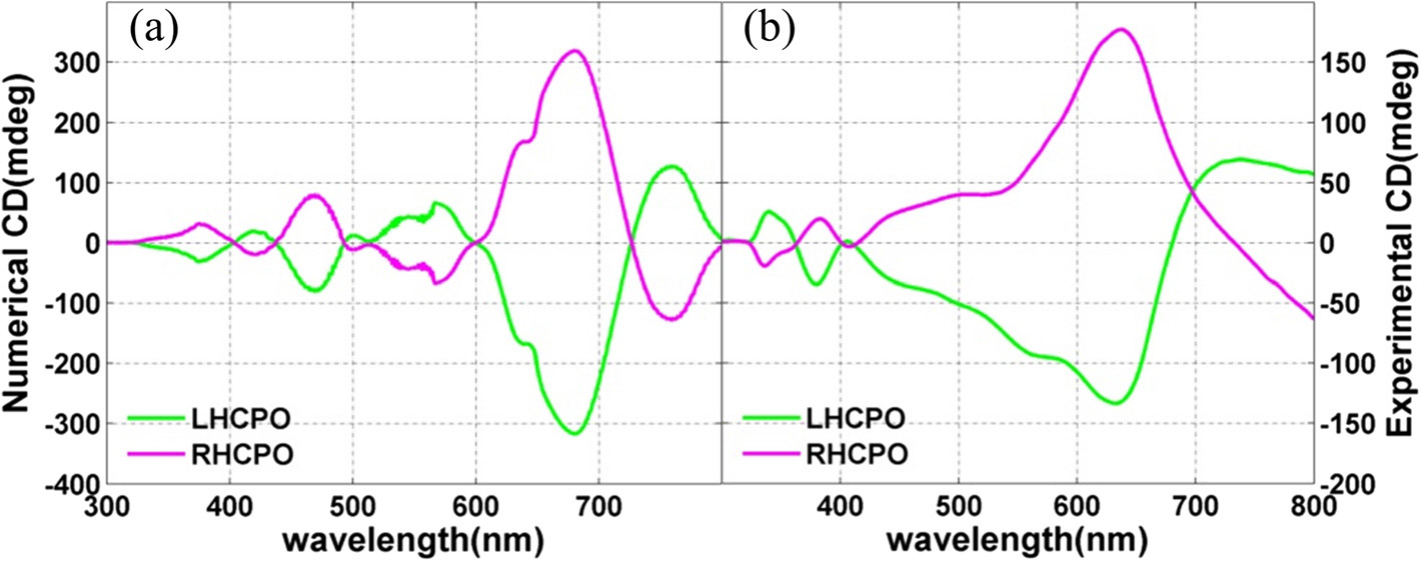
The simulated and measured CD spectra of LHCPO and RHCPO. **a** Calculated CD signal. **b** Measured CD

The finite difference time domain (FDTD) method is used to achieve the theoretical simulation and analysis of the obtained quasi-3D CPO. The geometric model of a single CPO used in simulation is four achiral particles with side length *L*_1, 2, 3, 4_ = 36, 96, 150, and 175 nm and thickness *k*_1, 2, 3, 4_ = 8, 25, 40, and 55 nm, respectively. These achiral particles are accurately arranged in a concentric circle like that shown in the obtained SEM images (i.e., Fig. 4a, b for LHCPO and RHCPO). Figure 5a shows the calculated CD spectra, where a maximum CD signal of about 320 mdeg is found at 680 nm. This obvious CD signal indicates that the CPO interact differently with LCP and RCP. Due to the small duty ratio of CPO on the glass substrate, the transmittance coefficient for LCP and RCP is larger than 0.70 in the whole visible range. These calculated results meet well with the CD spectra measured from the Jasco J-810 circular dichroism spectroscopy system (as shown in Fig. 5b), where the maximum CD signal is ~180 mdeg at 640 nm. The differences between the simulated and measured results should be mainly attributed to the geometric morphology difference of CPO used in theoretical simulation and experiment process.

**Fig. 6:**
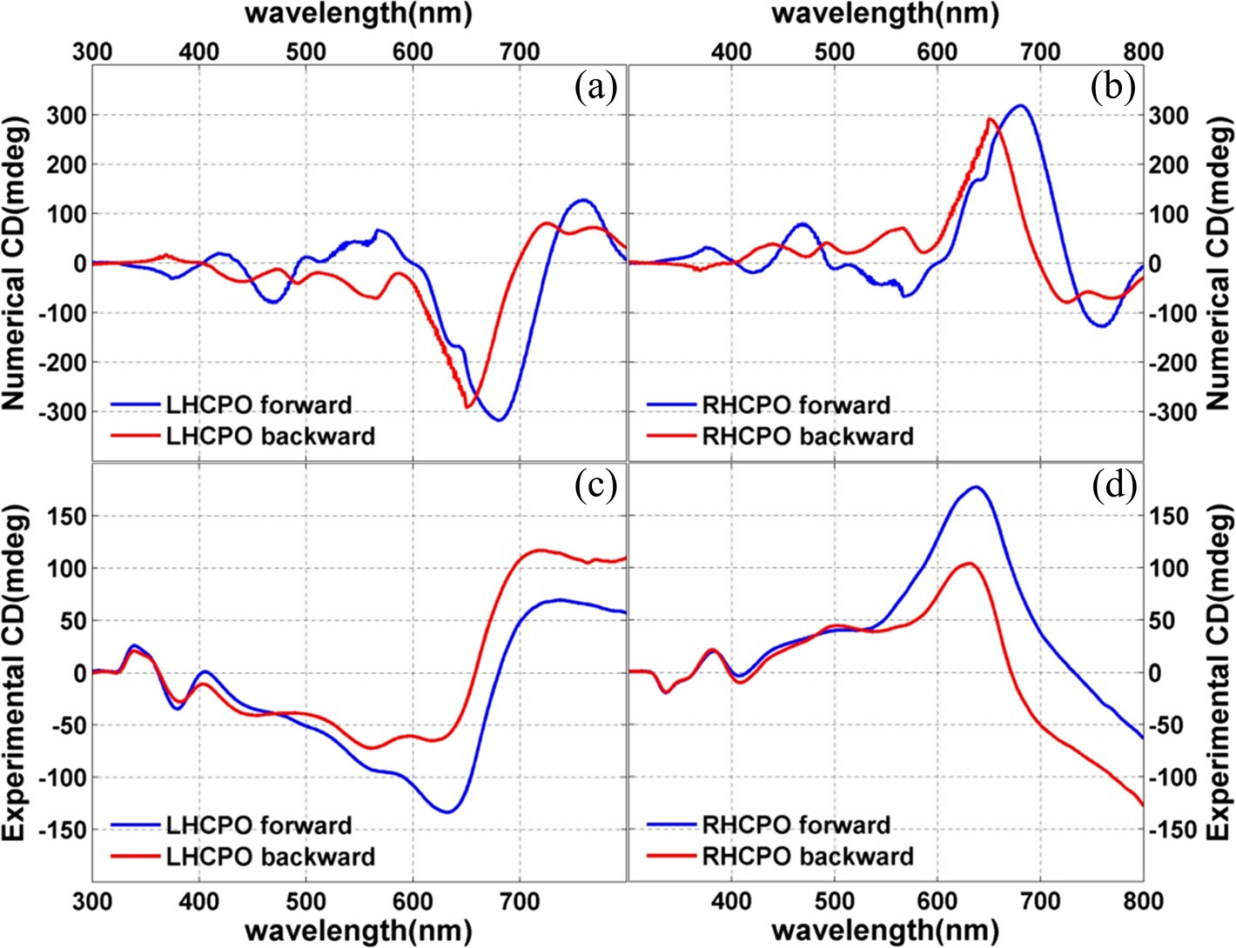
The simulated and experimental CD spectra. **a**, **b** Simulated CD spectra of LHCPO and RHCPO. **c**, **d** Measured CD spectra of LHCPO and RHCPO. The *blue* and *red curves* refer to the light incidence from the forward and backward sides of the samples

The geometric morphology plays an important role in determining the chiroptical effect. As discussed earlier, the chiroptical effect from 2D CPS usually is nonreciprocal, while the chiroptical effect from 3D CPS is reciprocal. This optical effect gives us a standard to determine the dimensionality of CPS. For the CPO obtained in this paper, it seems that they are a kind of 3D CPS from the point of view of geometric morphology. However, our in-depth theoretical and experimental researches reveal that the obtained CPO is indeed a kind of quasi-3D CPS, and their chiroptical effect is nonreciprocal, although they own a 3D geometrical morphology. Figure 6a, b shows the simulated CD spectra of LHCPO and RHCPO on the BK7 glass substrate when light incidents from the forward (blue curve) and backward (red curve) sides. The forward side is defined as the case that the BK7 glass substrate with the CPO side faces the light source, while the backward side is defined as the opposite case. These simulated results show that the CD signals from LHCPO and RHCPO own the same sign in the whole waveband from 300 nm to 800 nm, when light incidents from the forward and backward sides. These results meet well with the experiment, as shown in Fig. 6c, d. This 3D chiroptical effect indicates that the CPO together with BK7 glass substrate is a kind of 3D CPS.

**Fig. 7:**
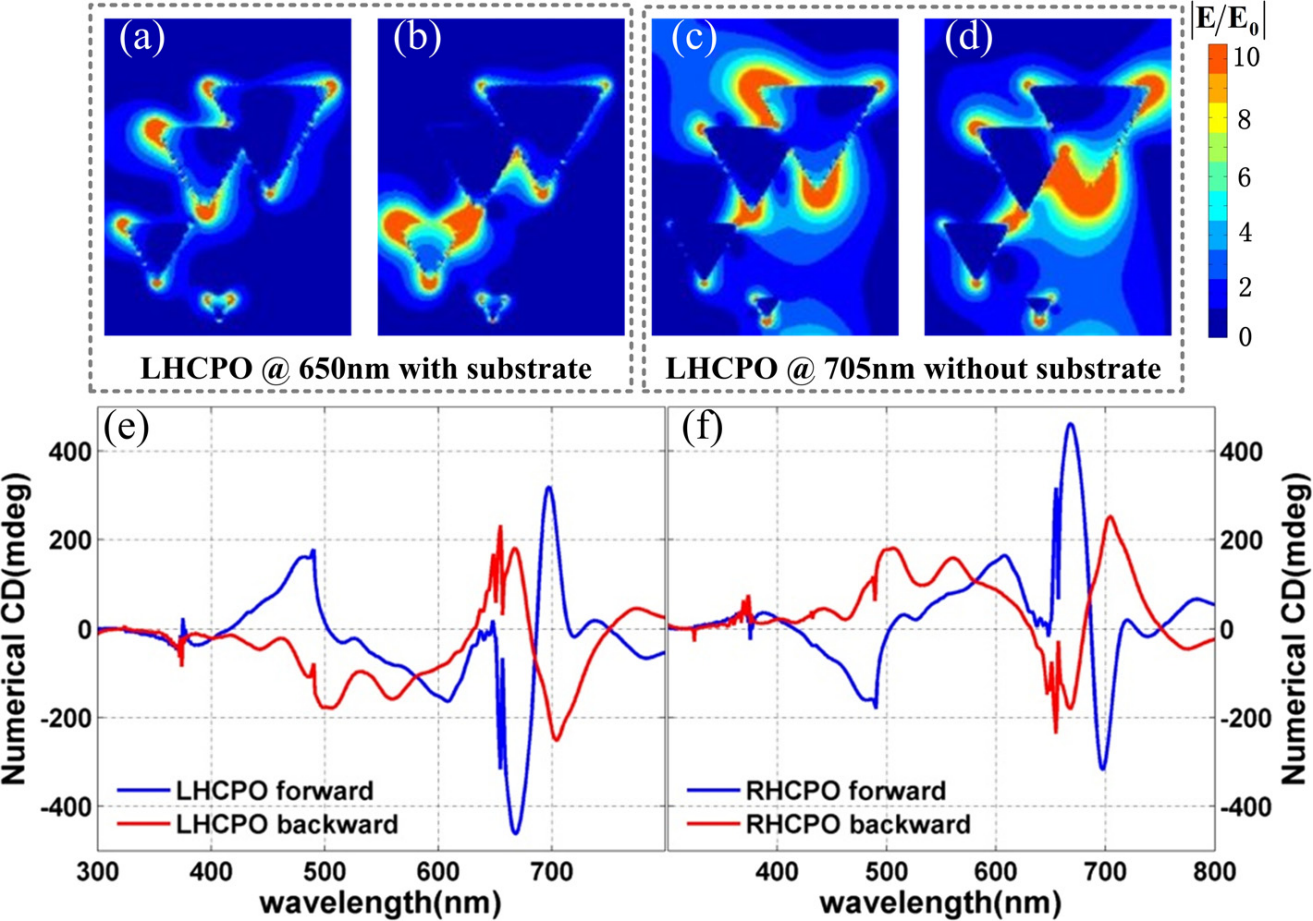
Images of the electric field distributions and simulated CD spectra of the CPO without substrate. **a**–**d** Electric field distribution at interface between CPO and BK7 glass substrate. **a**, **c** For LHCPO with substrate. **b**, **d** For LHCPO without substrate. The incident light is 650 nm for **a** and **b** and 705 nm for **c** and **d**. **e**, **f** Calculated CD spectra of LHCPO and RHCPO without substrate. The *blue* and *red curves* refer to the light incidence from the forward and backward sides

However, we note that the substrate also can affect the chiroptical effect from CPS. In fact, the excited surface plasmonic resonance in the air CPS and CPS substrate interfaces can destroy the mirror symmetry of 2D CPS and results in 3D chiroptical effect [37]. And this phenomenon happens again in this work. Figure 7e, f shows the simulated CD spectra of LHCPO and RHCPO without substrate when light incidents from the forward and backward sides. We can see that the CD signals almost own the opposite sign when light incidents from the opposite sides. This indicates that the chiroptical effect from CPO themselves is nonreciprocal, just like that from 2D CPS. And the 3D chiroptical effect shown in Fig. 6 should be attributed to the influence of the BK7 glass substrate, which adds another dimensionality for the quasi-3D CPO and results in reciprocal chiroptical effect. When comparing the simulated results in Fig. 6a, b with Fig. 7e, f, we find that the glass substrate also can suppress some plasmonic resonance modes and lead to a relatively smooth CD spectra shown in Fig. 6a, b. Figure 7a–d shows the surface electric distribution of LHCPO when LCP incidents from the backward side. Figure 7a, c is the electric distribution of LHCPO without substrate when incident lights are 650 and 705 nm, respectively. While Fig. 7b, d is the related electric distribution of LHCPO with substrate. These images clearly show that the BK7 glass substrate can effectively change the near-field distribution of LHCPO and finally the chiroptical effect. The CD intensity difference when light incidents from front and back sides (as shown in Fig. 7e, f) should be attributed to the influence of the 3D geometrical morphology of CPO, which is different when looking from the opposite sides.

**Fig. 8:**
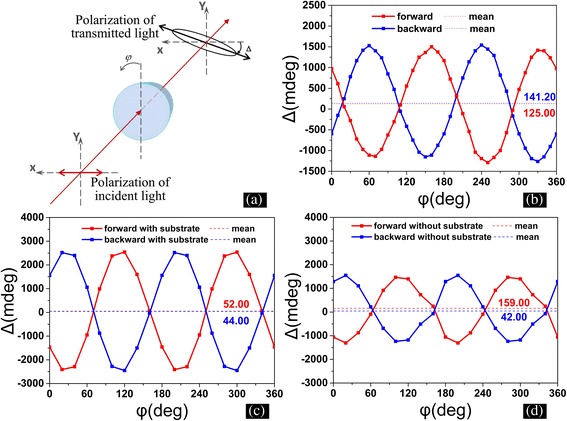
The experimental and simulated optical activity of LHCPO with wavelength of 632.8 nm. **a** Schematic illustration of the optical path used in the experiment. The angles *φ* and *Δ* refer to the incident polarization azimuthal angles of samples and polarization rotation angles, respectively. A He:Ne laser at 632.8 nm is used as a light source in the experiment, and the laser is replaced by a plane wave at 632.8 nm in simulations. **b** The measured polarization rotation angle *Δ* as a function of *φ* from LHCPO. **c**, **d** The simulated polarization rotation angle *Δ* as a function of *φ* from LHCPO with (**c**) and without substrate (**d**). The *blue* and *red colors* in **b**, **c**, and **d** refer to the light incidence from the backward and forward sides, while the *dashed lines* denote the averaged polarization rotation angles

The optical activity also has been investigated in this work. We note that the obtained CPO do not own a perfect in-plane rotational symmetry, and the polarization rotation of linear polarized light does not unambiguously determine the optical activity in the obtained CPO sample. Thus, the polarization analysis for linearly polarized incidence at a series of incident polarization azimuthal angles *φ* is performed to specify the linear birefringence and obtain the actual optical activity. The measured diagram is shown in Fig. 8a, where a linear polarization light at 632.8 nm incidents normally to the sample surface, and the polarization rotation angle *Δ* of the transmitted light is defined as the longer axis of the polarization ellipse with respect to the polarization direction of incident light. The measured result for LHCPO is shown in Fig. 8b, where the red and blue curves refer to the cases that light incidents from the forward and backward sides, respectively. The averaged polarization angles are 125.00 and 141.20 mdeg for the forward and backward incidences, respectively, which are indeed originated from the optical activity of LHCPO. Figure 8c shows the simulated polarization rotation angle *Δ* as a function of *φ* from LHCPO with substrate, and the averaged polarization angles are 52 and 44 mdeg for the forward and backward incidences, respectively, which meet well with the measured results shown in Fig. 8b. Figure 8d is the simulated polarization rotation angle *Δ* as a function of *φ* from LHCPO without substrate, where the averaged polarization rotation angles own the same sign. And this coincides well with the simulated CD signal shown in Fig. 7e, f, indicating the effectiveness of our previous discussions.

## Conclusions

In this paper, we have demonstrated that a kind of quasi-3D CPO can be easily achieved through the stepwise colloid sphere lithography technology. Through a four-step deposition process with proper fabrication parameters, the LHCPO and RHCPO are formed on a BK7 glass substrate with CD of ~180 mdeg and optical activity of ~140 mdeg. Due to the advantages of the fabrication method, the CPO with area of larger than 1 cm^2^ can be easily achieved, and their chiroptical resonance also can be expediently tuned in the infrared and visible region through using proper micro-sphere array and deposition parameters, such as deposition time, angle, and thickness. The FDTD method also has been employed to investigate the chiroptical properties of CPO, and the simulated results meet well with that of the experiment. The in-depth theoretical and experimental researches reveal that the BK7 glass substrate plays an important role in the chiroptical effect of CPO. The substrate can restrain some plasmonic resonance modes and obviously break the mirror symmetry of CPO, leading to a 3D chiroptical effect in the experiment. The CPO with large area, tunable chiroptical spectra, and a low-cost fabrication method could find potential applications in many fields, such as stereochemical enantiomer sensors.

## Abbreviations

2D: two-dimensional
3D: three-dimensional
AFM: atomic force microscopy
CD: circular dichroism
CPO: chiral plasmonic oligomers
CPS: chiral plasmonic structures
FDTD: finite difference time domain method
LCP: left circular polarization
LHCPO: left-handed chiral plasmonic oligomers
PDMS: polydimethylsiloxane
PS: polystyrene
RCP: right circular polarization
RHCPO: right-handed chiral plasmonic oligomers
SEM: scanning electron microscopy
